# Ifosfamide, Carboplatin, and Etoposide (ICE) in Combination with Regional Hyperthermia as Salvage Therapy in Patients with Locally Advanced Nonmetastatic and Metastatic Soft-Tissue Sarcoma

**DOI:** 10.1155/2020/6901678

**Published:** 2020-02-27

**Authors:** Veit Bücklein, Christina Limmroth, Eric Kampmann, Gesa Schuebbe, Rolf Issels, Falk Roeder, Martin Angele, Hans Roland Dürr, Thomas Knösel, Sultan Abdel-Rahman, Dorit Di Gioia, Lars H. Lindner

**Affiliations:** ^1^Department of Medicine III, University Hospital, LMU Munich, Munich, Germany; ^2^Medizinische Klinik Köln-Holweide, Cologne, Germany; ^3^Department of Radiotherapy and Radio-Oncology, Landeskrankenhaus, Paracelsus Medical University Clinics, Salzburg, Austria; ^4^Department of General, Visceral, and Transplant Surgery, University Hospital, LMU Munich, Munich, Germany; ^5^Department of Orthopedics, University Hospital, LMU Munich, Munich, Germany; ^6^Institute of Pathology, University Hospital, LMU Munich, Munich, Germany

## Abstract

Patients with localized relapse of soft-tissue sarcoma (STS) after anthracycline-based chemotherapy have a dismal prognosis, particularly when surgery is not possible. To facilitate resection and improve long-term tumor control, we applied an intensified perioperative treatment consisting of ICE (ifosfamide 6 g/m^2^, carboplatin 400 mg/m^2^, and etoposide 600 mg/m^2^) in combination with regional hyperthermia (RHT) to maximize local control. Here, we retrospectively evaluate the safety and efficacy of this strategy. Patients aged ≥18 years with locally advanced high-risk STS, either with or without metastasis, treated with ICE + RHT after the failure of first-line anthracycline-based chemotherapy were included in this analysis. Radiographic response, toxicity, progression-free survival (PFS), and overall survival (OS) were assessed. Between 1996 and 2018, 213 sarcoma patients received ICE at our centre. Of these, 110 patients met the selection criteria (progressive disease, suitable high-grade STS histology, anthracycline pretreatment, RHT treatment) for this analysis. Fifty-four patients had locally advanced disease without metastases (LA-STS), and 56 patients had additional metastatic disease (M-STS). Disease control was achieved in 59% of LA-STS patients and in 47% of M-STS patients. For LA-STS, 21% of the patients achieved radiographic response, facilitating resection in 4 patients (7%), compared with 11% of the M-STS patients, facilitating resection in 5 patients (9%). PFS was significantly longer in LA-STS than in M-STS (10 vs. 4 months, *p* < 0.0001). Median OS was 26 months in LA-STS and 12 months in M-STS. Disease control was the only independent prognostic factor for improved OS in multivariate analysis. Toxicity was high with neutropenic fever occurring in 25% of the patients and three therapy-related deaths (3%). ICE + RHT demonstrated activity in high-risk STS and facilitated resection in selected patients after anthracycline failure. Disease control was associated with improved OS. Based on the observed toxicities, the dose should be reduced to 75%.

## 1. Introduction

In the ongoing efforts to improve the outcome of patients suffering from soft-tissue sarcoma (STS), the management of local tumor relapse after intensive multimodal first-line therapy is rarely addressed [1]. For all STS patients, local relapse occurs in approximately 20% of patients [2–5], but these rates can be significantly higher in patients with insufficient resection margins and/or high-risk disease [4, 6, 7]. Patients with recurring STS have a disappointing prognosis [4, 8, 9], illustrating the need for additional therapeutic options. This is particularly true for patients after failure of prior (neo)adjuvant chemo- and/or radiotherapy applied in high-risk situations.

Multimodal treatment including (neo)adjuvant anthracycline/ifosfamide-based chemotherapy in combination with regional hyperthermia (RHT) can improve progression-free and overall survival of STS patients with localized disease when compared with (neo)adjuvant chemotherapy alone [10]. Furthermore, response rates are doubled by the addition of RHT [7] and are higher than agents approved for use in the second-line treatment of STS (such as trabectedin, eribulin, or pazopanib) [11–14]. For patients with non- or borderline-resectable relapse of STS, intensification of local therapy by combining an anthracycline-free polychemotherapy with RHT might therefore be a promising therapeutic approach.

Here, for the first time, we report the outcomes of such an intensified multimodal therapeutic strategy, after anthracycline failure, in a larger cohort of patients. We retrospectively analyzed the safety, tolerability, and efficacy of a polychemotherapy consisting of ifosfamide, carboplatin, and etoposide (ICE) in combination with RHT in patients with relapsed/refractory STS. We included patients with local relapse or progression of their tumor after anthracycline-based chemotherapy. One subcohort encompassed patients without radiographic evidence of metastases (LA-STS), whereas patients with predominant local relapse and concomitant metastatic disease were analyzed as a separate patient cohort (M-STS).

## 2. Patients and Methods

### 2.1. Patients

Patients aged ≥18 years treated with ICE + RHT with a histological diagnosis of locally advanced high-risk STS, and progression or relapse of their disease after prior anthracycline-based chemotherapy, were included in this retrospective analysis. We divided the patients according to the presence or absence of metastases: patients with evidence of locally advanced STS without metastases (LA-STS) were analyzed separately from patients with metastatic disease (M-STS). All patients with metastatic disease, however, also suffered from local, RHT-targetable relapse of the STS. Evaluated histologic subentities for both populations encompassed adult-type STS according to the World Health Organization (WHO) classification of tumors [15]. Patients with gastrointestinal stromal tumors, clear cell sarcoma, angiosarcoma, and desmoplastic small round cell tumors were excluded due to differences in management and course of the disease.

Only patients with high-risk disease (defined as Fédération Nationale des Centres de Lutte Contre Le Cancer (FNCLCC) grade 2 or 3, tumor diameter ≥5 cm, located deep to the fascia) with radiographic evidence of disease progression were selected for the analysis. Patients who had not received an anthracycline-containing pretreatment or had not been treated with RHT were excluded. The analysis was approved by the local Institutional Review Board on Medical Ethics, and the Declaration of Helsinki was observed.

### 2.2. Treatment

ICE chemotherapy consisted of ifosfamide (1,500 mg/m^2^, days 1–4), carboplatin (100 mg/m^2^, days 1–4), and etoposide (150 mg/m^2^, days 1–4). Treatment was repeated on day 28 until disease progression or for up to 8 cycles. Granulocyte-colony stimulating factor (G-CSF) support was mandatory. In case of insufficient blood count recovery, treatment was delayed. Dose reductions were applied if severe thrombocytopenia or neutropenic fever occurred, or in case of repeated treatment delays.

RHT, performed according to published quality and safety guidelines [16], was applied on days 1 and 3 or on days 1 and 4 of each ICE cycle. We used the BSD-2000 hyperthermia system (BSD Medical Corporation, Salt Lake City, UT, USA) to induce tumor temperatures of 40–43°C for 60 minutes. Ifosfamide was infused during RHT treatment. The RHT field was focused to the tumor area. For patients with metastatic disease, locoregional metastases (if present) were included in RHT field.

### 2.3. Imaging

Staging procedures encompassed computerized tomography (CT) scans for all patients, and magnetic resonance imaging (MRI) scans for patients with extremity tumors, and were usually performed after three, six, and eight cycles of treatment. After completion of treatment, radiographic reevaluation was performed every three months for two years, and every six months thereafter. Response was assessed according to Response Evaluation Criteria in Solid Tumors (RECIST) 1.1 [17]. Only subjects with measurable disease at the time of treatment initiation were included in the response assessment. For patients receiving tumor resection, radiographic response was given for the latest time point prior to resection.

### 2.4. Assessment of Hematological Toxicity and Infectious Complications

Due to the retrospective nature of the analysis, data on nonhematologic toxicity were limited. Frequencies of hematologic toxicity, however, were well documented, as grade IV toxicities (according to Common Terminology Criteria for Adverse Events [CTCAE] 4.03 criteria [18]) and infectious complications generally lead to dose reductions.

### 2.5. Statistical Analyses

To evaluate the efficacy of ICE + RHT, the progression-free rate (PFR) three and six months after the first administration of ICE + RHT was assessed in patients with metastatic disease (M-STS), with particular focus on patients who did not undergo tumor resection in the context of ICE + RHT. We focused on this subcohort to evaluate the efficacy of ICE + RHT, as these patients best resembled the patient population included in the publication of Glabbeke et al. [19], in which reference values for effective second-line treatment regimens for patients with STS have been determined.

Three- and six-month PFS were calculated for all patients (in both the LA-STS and M-STS cohorts) without clinical or radiographic evidence of progression who were alive three and six months after treatment initiation, respectively. PFS and OS were calculated according to the Kaplan–Meier method, and 95% confidence intervals (CI) were estimated according to Greenwood's formula [20]. Patients without confirmed progression, relapse, or death after ICE + RHT treatment were censored at the time of the last assessment. Differences in survival were assessed by the stratified log-rank test. A two-sided *p* ≤ 0.05 was used to determine significance. Multivariate analyses were calculated using the Cox model. All statistical analyses were conducted using R (version 3.5.2) with the “Survival” package (Version 2.35) [21].

## 3. Results and Discussion

### 3.1. Patients' Characteristics

Between 1996 and 2018, 213 sarcoma patients were treated with ICE. Of these, 110 patients met the selection criteria and were included in the analysis. The LA-STS subcohort consisted of 54 patients, whereas 56 patients were included in the M-STS subgroup with metastatic disease (Supplementary [Supplementary-material supplementary-material-1]). Patients' characteristics are given in Table 1. Of note, ICE + RHT was applied as postoperative consolidation regimen after resection of relapse in 10 (18.5%) LA-STS and 1 (1.8%) M-STS patients, respectively (Table 2).

### 3.2. Treatment

In LA-STS patients, a median of four cycles of ICE chemotherapy were applied, together with a median of seven RHT treatments. In M-STS patients, the median of ICE cycles was also four, applied in combination with a median of six RHT treatments.

For all patients, particularly in the LA-STS subcohort of patients, (re-)resections and/or radiotherapeutic options were regularly reevaluated over the course of the therapy. Procedures shown in Table 2 encompass surgical treatments and radiotherapy applied in between cycles or after completion of ICE + RHT.

**Figure 1:**
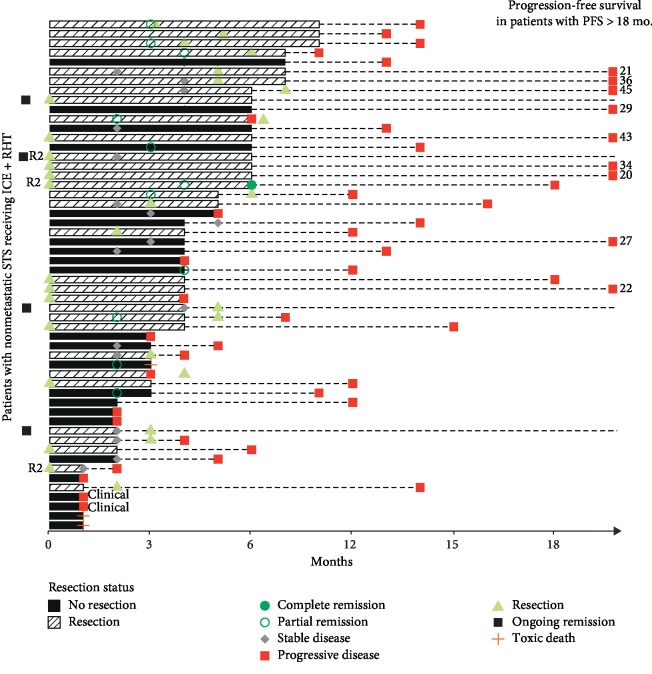
Swimmer plot depicting the clinical course of LA-STS patients (*n* = 54). Bars represent the treatment duration of ICE + RHT (range: 1–8 months). Results of radiographic assessments and other major clinical events are represented by different symbols, as depicted on the bottom. mo., months and PFS, progression-free survival.

Details on ICE + RHT treatment duration, imaging results, and surgical procedures in LA-STS patients are depicted in Figure 1. Of note, tumor resection was performed in 31/54 (57%) of LA-STS patients and in 15/56 (27%) of M-STS patients. Complete resection with adequate margins (R0) was possible in 13% (7/54) of all patients without metastases and in 9% (5/56) of all patients with metastatic disease (including metastasectomy), respectively. R1 and R2 resections were performed in 10/54 (19%) and 10/54 (19%) LA-STS patients and in 4/56 (7%) and 4/56 (7%) M-STS patients, respectively. The resection margin status remained unclear (Rx) in 4/54 (7%) LA-STS and 2/56 (4%) M-STS patients. In nine patients with radiographic response (8% of all treated patients), tumor resection was facilitated by ICE + RHT (four in LA-STS and five in M-STS patients, respectively).

### 3.3. Response to Treatment

For radiographic response, 40 patients could be evaluated in the LA-STS subcohort population, as ten patients had received ICE + RHT as postoperative consolidation regimen and had no measurable disease prior to ICE + RHT. No radiographic response assessment was performed in nine LA-STS patients due to clinical evidence of early disease progression in two patients, therapy-related infectious deaths in two patients, and discontinuation of treatment without response assessment in five patients. In the M-STS subcohort, one patient was not evaluable for response due to resection (including metastasectomy) prior to ICE + RHT. In four patients, no radiographic response assessment was possible because of early clinical progression of the disease, and one patient discontinued treatment without response assessment. Results are given in Table 3. Objective responses were seen in 20% (9/44) of LA-STS patients, compared with 11% (6/55) in M-STS patients. Disease control was obtained in 59% (26/44) and 47% (26/55) of the LA-STS and M-STS patients, respectively. Two patients with stable disease (SD) and three patients with initial partial remission (PR) progressed over continued treatment with ICE + RHT in the LA-STS cohort, whereas only one patient with initial PR achieved a (short-lasting) complete remission (CR) with further ICE + RHT therapy. In the M-STS subcohort, three patients with initial PR and eight patients with initial SD had progression of their STS with ongoing ICE + RHT treatment.

### 3.4. Survival

Median follow-up was 38 months (range 0–188 months) for all censored patients (*n* = 12) in the LA-STS subcohort at time of analysis, and median observation time of all analyzed LA-STS patients was 23 months. For patients with metastatic disease (M-STS), median follow-up of all censored patients (*n* = 6) and median observation time was 7 and 11 months, respectively.

#### 3.4.1. Progression-Free Survival

Four of the 54 patients in the LA-STS subgroup (7%) remained progression-free following multimodal treatment. Out of these four patients, ICE + RHT was applied as postoperative regimen in two patients and as preoperative regimen in two patients. Within the subgroup of patients with metastatic disease, progression of disease occurred in all 56 patients.

Progression-free rates (PFR) after 3 and 6 months are given in Table 4. Patients with metastatic disease without surgical treatment, the patient subgroup that best resembles the reference population from the original publication of Glabbeke et al. [19], had a PFR of 41% after three and 20% after six months, indicating activity of the regimen.

**Figure 2:**
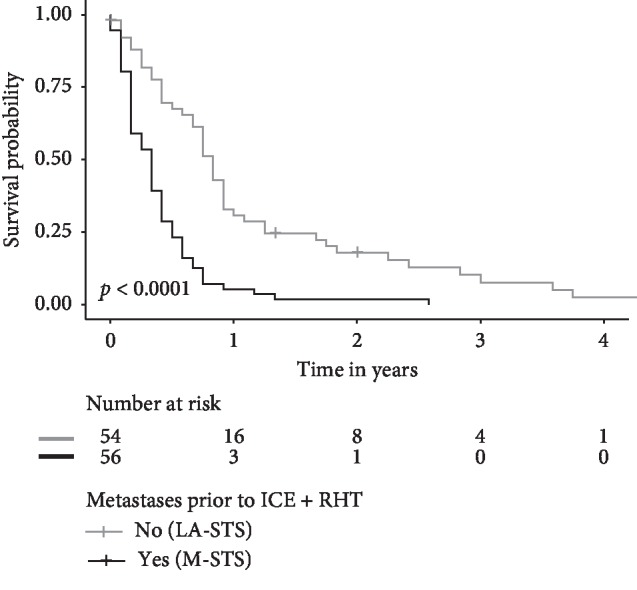
Kaplan–Meier estimates of progression-free survival for patients with (*n* = 56, M-STS) and without (*n* = 54, LA-STS) metastases prior to initiation of ICE + RHT. The *p* value was assessed by the log-rank test.

Median progression-free survival (PFS) was 10 months for all patients in the LA-STS population (95% CI 8–11 months). It was significantly shorter for patients with metastatic disease (M-STS, 4 months [95% CI 2–5 months], *p* < 0.0001, Figure 2). This was due to early progression of distant metastasis, as *local* PFS was not significantly different between both patient groups (6 months [95% CI 4–8 months] for M-STS patients vs. 11 months [95% CI 9–13 months] for LA-STS patients, *p*=0.185, data not shown).

For patients with nonmetastatic disease (LA-STS), median PFS was independent of age (>50 years vs. ≤50 years), sex, and treatment line (second-line treatment vs. >second-line treatment; data not shown). In univariate analysis, grading, histologic subtype (lipo/leiomyosarcoma vs. other subentities), type of progression (primary refractory disease vs. progression after response to prior chemotherapy), response to ICE + RHT (SD/PR/CR vs. PD), and surgery significantly influenced progression-free survival. In multivariate analysis, grading, type of relapse, and response to therapy remained of significant influence (Supplementary [Supplementary-material supplementary-material-1]).

For M-STS patients, response to ICE + RHT (SD/PR/CR vs. PD) was the only factor significantly influencing PFS in univariate (*p* < 0.0001) and multivariate analyses (*p* < 0.001, data not shown).

#### 3.4.2. Overall Survival

**Figure 3:**
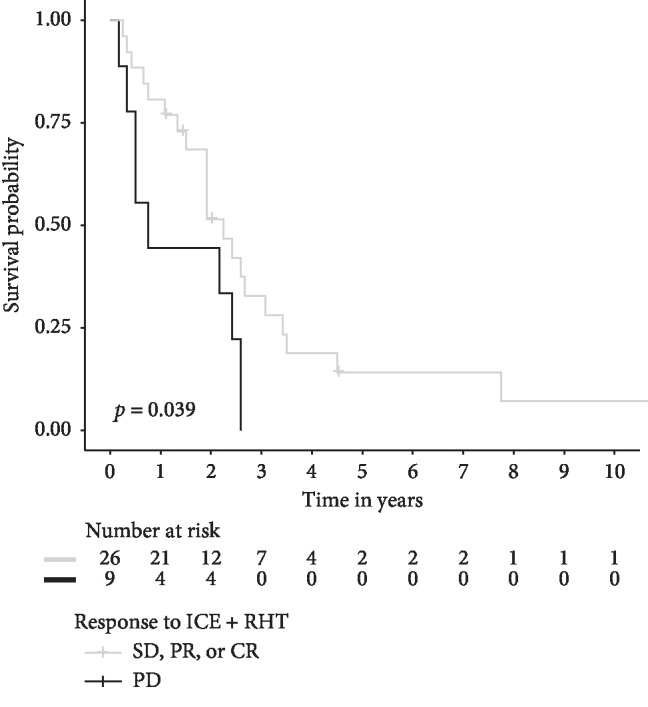
Kaplan–Meier estimates of overall survival for LA-STS patients with disease stabilization or response (SD/PR/CR, *n* = 26) and progressive disease (PD, *n* = 9) as the best radiographic response to ICE + RHT. CR, complete response; SD, stable disease; PD, progressive disease; and PR, partial remission. The *p* value was assessed by the log-rank test.

Median overall survival for the LA-STS population was 26 months (95% CI 18–31 months). Patients with metastatic disease (M-STS) had a significantly shorter overall survival (12 months, 95% CI 10–17 months, *p*=0.002; data not shown). Factors significantly influencing overall survival in the univariate analysis for LA-STS patients were surgical resection of the tumor, type of progression (primary refractory disease vs. progression after response to prior chemotherapy), and response to ICE + RHT (SD/PR/CR vs. PD, 27 vs. 9 months, *p*=0.039, Figure 3). The latter remained the only independent factor influencing overall survival in the multivariate analysis (Table 5).

Similar to PFS, response to ICE + RHT (SD/PR/CR vs. PD) remained the only factor significantly influencing OS in the univariate (*p* < 0.0001) and multivariate analysis (*p*=0.002) for M-STS patients (Supplementary [Supplementary-material supplementary-material-1]).

### 3.5. Hematological Toxicity, Dose Reductions, and Infectious Complications

Hematological toxicity was common after treatment with ICE + RHT. Of all treated patients, almost two-thirds showed grade III or IV toxicity according to the CTCAE V4.03 criteria (Table 6). Therapy-induced neutropenia lead to infectious complications with fever in 27 of the 110 treated patients (24.5%; 15/54 in LA-STS and 12/56 in M-STS). Three LA-STS patients died due to therapy-related infections (6% of LA-STS patients or 3% of all patients, Table 6).

Dose reductions were frequent. In the majority of patients, applying a full-dosed ICE cycle was never deemed feasible. Further dose reductions were needed in more than 50% of all patients, including patients that never received a full-dosed ICE cycle. Relative dose intensities are reported in Supplementary [Supplementary-material supplementary-material-1].

## 4. Discussion

Prognosis of patients with STS has shown little improvement over the past decades [14]. Wide tumor resection remains standard of care. In patients with high-risk disease (G2/3, tumor diameter ≥5 cm, location deep to the fascia), treatment is regularly intensified by radiotherapy and anthracycline-containing chemotherapy.

However, relapse after resection, even after intensified pretreatment, occurs frequently, particularly in patients with retroperitoneal or visceral location of their tumor. When relapse is limited to locoregional recurrence of the tumor, a curative approach is appropriate. Even with oligometastatic disease, metastasectomy should be considered, as metastatic spread does not preclude durable remissions after successful resection [22]. However, no evidence-based treatment strategy has been outlined for patients in this situation so far [1], and there is a clear medical need to improve the outcome.

Here, we report results of a multimodal therapy approach for patients with failure of a previous anthracycline-containing chemotherapy. The multimodal approach included RHT to maximize the local efficacy of chemotherapy. RHT, when applied in parallel, improves response rates of chemotherapy in high-risk STS, compared with chemotherapy alone [10], and might therefore facilitate resection in otherwise nonresectable STS. ICE, a polychemotherapy regimen reportedly active in smaller series of STS patients [23, 24], was used. To our knowledge, our analysis is the first to report results of an intensified perioperative chemotherapy approach in the context of relapsed or refractory STS. In addition to the LA-STS and M-STS general analyses, we also separately analyzed the outcomes of a subcohort with metastatic STS (M-STS), treated with ICE + RHT at our institution over the past years to estimate the efficacy of the treatment. All patients in this subcohort had predominant local relapse and therefore received chemotherapy in combination with RHT. Nevertheless, the M-STS population closely resembled the patient populations used by Glabbeke et al. to establish reference values to determine efficacy in anthracycline-refractory STS [19]. Estimation of PFR three months after initiation of therapy with ICE + RHT in the M-STS subcohort showed ongoing response to treatment or progression arrest in 54% of the patients, indicative of an active treatment regimen (determined to induce a PFR ≥ 40% three months after initiation of treatment) per the criteria proposed by Glabbeke et al. PFR after three months in patients with metastatic disease who did not receive tumor resection also met the criteria of activity (41%, Table 4).

Response rates were higher in LA-STS patients than in the M-STS population (21% vs. 11%, Table 2), comparing favorably with single-agent therapies applied in anthracycline-refractory STS [11, 12, 25]. Interestingly, progression-free survival was shorter in M-STS patients (4 vs. 10 months, Figure 2) due to progression of metastasis. Local progression-free survival was not significantly different between LA-STS and M-STS patients, hinting at the contribution of RHT treatment to an improved disease control.

Response to ICE + RHT treatment facilitated resection in a subgroup of patients, which accounted for 7% of the LA-STS population without metastases and 9% of the M-STS population. Tumor resection is a contributor with borderline significance in multivariate analyses for progression-free and overall survival in the LA-STS subcohort, and ICE + RHT might therefore lead to improved survival for patients with otherwise dismal prognosis due to the impossibility of sufficient tumor resection. The strongest evidence for the favorable activity of ICE + RHT, however, derives from the observation that disease stabilization, or CR, or PR with ICE + RHT was associated with improved OS in the univariate analysis (Figure 3), which remained the only significant prognostic factor for improved overall survival in the multivariate analysis (Table 5) for both LA-STS and M-STS patients. Recently, Grünwald et al. demonstrated that the absence of disease progression is the main factor predicting therapeutic efficacy of anthracycline-based chemotherapy in patients with advanced STS [26]. This is in line with our findings, highlighting the major importance of disease control for improved outcome in STS.

Response kinetics show that the best response is usually achieved within two to four cycles of treatment, as only one patient with a PR after four cycles achieved a deeper remission (CR) after six cycles of chemotherapy within the LA-STS subcohort. In contrast, progression of disease during ongoing ICE + RHT treatment in patients with initially stable or responding STS occurred in five patients in the LA-STS subcohort and eleven patients in the M-STS subcohort. These observations hint at the necessity of early re-resection of the tumor after two to four cycles of therapy.

As progression-free and overall survival were independent of STS subentities (lipo-/leiomyosarcoma vs. other subentities) in the multivariate analysis, ICE + RHT can be considered as salvage therapy in a wide range of STS histologies (Table 5 and Supplementary [Supplementary-material supplementary-material-1]).

However, this favorable antitumor activity is associated with relevant toxicity. Grade III/IV hematological toxicity was observed in more than 60% of all patients, despite dose reductions in more than 50% of all patients. Neutropenic fever frequently occurred and lead to three sepsis-related deaths. Compared with other chemotherapeutic regimens used in the second-line treatment of STS, hematologic toxicity and infectious complications are common with ICE + RHT [11, 12, 27–29]. We therefore adapted the starting dose of ICE + RHT and recommend a starting dose of 75% of the originally intended dose (cumulative doses per cycle: ifosfamide 4.5 g/m^2^, carboplatin 300 mg/m^2^, and etoposide 450 mg/m^2^).

## 5. Conclusions

ICE + RHT is an active salvage therapy regimen in the treatment of STS patients after the failure of an anthracycline-containing pretreatment. In patients with nonresectable localized relapse or with progression during anthracycline-based chemotherapy, ICE + RHT leads to disease stabilization in a significant proportion of patients and can facilitate resection in a subgroup of patients.

The use of ICE + RHT in patients with locally advanced relapse or progression under anthracycline-based pretreatment should therefore be evaluated in prospective clinical trials. Patients planned for re-resection should receive no more than four cycles of treatment, as patients without radiographic evidence of response are not expected to respond later on, and patients with initial stable or responding STS may have subsequent disease progression. If resectability cannot be reached, treatment with ICE + RHT should be continued nevertheless, as disease stabilization and response are associated with improved OS in these patients with otherwise dismal prognosis.

## Figures and Tables

**Table 1:** Patients' characteristics and pretreatment. *p* values, representing differences between patients with locally advanced disease and patients with metastatic disease, were assessed by Fisher's exact test for sex, progression vs. relapse, grading, and pretreatment procedures, by the chi-square test for anatomic site and histopathologic subtype, and by the unpaired *t*-test for age.

	Locally advanced disease	Metastatic disease	*p*
	(LA-STS, *n* = 54) No. (%)	(M-STS, *n* = 56) No. (%)	
Age			0.23
Median (range)	56 (18–74) years	51 (21–77) years	
<51 years	16 (29.6)	27 (48.2)	
>50 years	38 (70.4)	29 (51.8)	

Sex			0.26
Male	31 (57.4)	26 (46.4)	
Female	23 (42.6)	30 (53.6)	

Progression vs. relapse			**0.01**
Progression of pre-existing lesion(s)	22 (40.7)	37 (66.1)	
Relapse after disease-free interval	32 (59.3)	19 (33.9)	
Median (range) duration of the disease-free interval	27 (9–171) months	14 (2–115) months	

Anatomic site			0.16
Head and neck	2 (3.7)	1 (1.8)	
Extremities and trunk wall	23 (42.6)	30 (53.6)	
Extremities	18 (33.3)	17 (30.4)	
Trunk wall	5 (9.3)	13 (23.2)	
Trunk	29 (53.7)	25 (44.6)	
Abdomen	10 (18.5)	15 (26.8)	
Retroperitoneum	13 (24.1)	6 (10.7)	
Pelvis	6 (11.1)	4 (7.1)	

Site of metastasis (multiple sites possible)			
Lung		35 (62.5)	
Liver		13 (23.2)	
Nodal		8 (14.3)	
Soft tissue		7 (12.5)	
Osseous		3 (5.4)	
Other		8 (14.3)	

Histopathologic subtype			0.051
Liposarcoma	20 (37.0)	6 (10.7)	
Myxoid	2 (3.7)	0 (0.0)	
Dedifferentiated	18 (33.3)	6 (10.7)	
Leiomyosarcoma	8 (14.8)	16 (28.6)	
Rhabdomyosarcoma	2 (3.7)	4 (7.1)	
Undifferentiated pleomorphic sarcoma (UPS)	11 (20.4)	14 (25.0)	
Synovial sarcoma	1 (1.9)	3 (5.4)	
MPNST	5 (9.2)	4 (7.1)	
Other	7 (13.0)	9 (16.1)	

FNCLCC grading			0.32
Grade 2	22 (40.7)	17 (30.4)	
Grade 3	32 (59.3)	39 (69.6)	

Pretreatment			
1 regimen	46 (85.2)	40 (71.4)	0.11
>1 regimen	8 (14.8)	16 (28.6)	
Anthracycline-containing	54 (100.0)	56 (100.0)	
Ifosfamide-containing	52 (96.3)	55 (98.2)	
Surgery	48 (88.9)	45 (80.4)	0.29
Radiotherapy	31 (57.4)	18 (32.1)	**0.02**

MPNST: malignant peripheral nerve sheath tumor.

**Table 2:** ICE + RHT treatment characteristics.

	Locally advanced disease (LA-STS, *n* = 54)	Metastatic disease (M-STS, *n* = 56)
	Median (range)	Median (range)
ICE cycles	4 (1–8)	4 (1–8)
RHT treatments	8 (2–16)	6 (1–16)
	No. of patients (%)	No. of patients (%)
Radiotherapy	6 (11.1)	6 (10.7)
Surgery	31 (57.4)	15 (26.8)
Surgery prior to start of ICE + RHT	10 (18.5)	1 (1.8)
R0	7 (13.0)	5 (8.8)
R1	10 (18.5)	4 (7.2)
R2	10 (18.5)	4 (7.2)
Rx	4 (7.4)	2 (3.6)
Including metastasectomy		8 (14.3)
Excluding metastasectomy		7 (12.5)

R0, complete resection of the tumor, with no microscopic evidence of tumor infiltration of resection margins; R1, complete macroscopic resection of the tumor, with microscopic evidence of tumor infiltration of resection margins; R2, incomplete resection of the tumor, with macroscopic tumor burden remaining in situ; Rx, resection with unknown resection margins.

**Table 3:** Radiographic response to ICE + RHT treatment. For patients receiving tumor resection during or directly after ICE + RHT, radiographic response is given for the latest time point prior to resection. Percentages of CR, PR, NC and PD are given as proportions of patients with measurable disease.

	Locally advanced disease (LA-STS, *n* = 54)	Metastatic disease (M-STS, *n* = 56)
	No. (%)	No. (%)
No measurable disease	10 (18.5)	1 (1.8)
Measurable disease	44 (81.5)	55 (98.2)
CR	1 (2.3)	0 (0.0)
PR	8 (18.2)	6 (10.9)
SD	17 (38.5)	20 (36.4)
PD	9 (20.5)	24 (43.6)
Could not be evaluated	9 (20.5)	5 (9.1)
Objective response	9 (20.5)	6 (10.9)
Disease control	26 (59.1)	26 (47.3)

CR, complete response; SD, stable disease; PD, progressive disease; PR, partial response.

**Table 4:** Progression-free survival rates 3 and 6 months after initiation of ICE + RHT.

Patient subpopulation	PFR after 3 months (95% CI)	PFR after 6 months (95% CI)
Patients with nonmetastatic disease (*n* = 54, LA-STS)	82% (72–93%)	67% (56–82%)
Patients with metastatic disease (n = 56, M-STS)	54% (42–68%)	23% (14–37%)
Patients with metastatic disease without surgical treatment (*n* = 41)	41% (29–60%)	20% (10–36%)

**Table 5:** Prognostic factors for overall survival for LA-STS patients. *p* values were calculated using the log-rank test for univariate analysis. For multivariate analysis using a Cox model, only parameters with a *p* < 0.10 in univariate analyses were considered.

Parameter	*N*	Univariate analysis		Cox model	
		Median OS, mo. (95% CI)	*p*	HR (95% CI)	*p*
Age			0.46		
>50 years	38	26 (18–40)			
≤50 years	16	23 (10–41)			

Sex			0.57		
Male	31	28 (13–41)			
Female	23	23 (18–93)			

Treatment line			0.31		
2nd line	46	26 (18–37)			
>2nd line	8	20 (10-NA)			

Grading			0.12		
3	32	23 (16–31)			
2	22	31 (28-NA)			

Histologic subentity			0.23		
Lipo-/leiomyosarcoma	28	29 (13–93)			
Non-lipo-/leiomyosarcoma	26	23 (18–37)			

Surgery			**0.014**	0.52 (0.25–1.10)	0.088
Yes	31	18 (8–31)			
No	23	28 (23–93)			

Response/disease stabilization as the best radiographic response with ICE + RHT			**0.039**	0.42 (0.18–0.96)	**0.041**
Yes	26	27 (23–41)			
No	9	9 (6–NA)			

Type of progression			**<0.001**	0.69 (0.33–1.45)	0.332
Progression after response to prior chemotherapy	32	31 (23–94)			
Primary refractory disease	22	13 (9–31)			

HR, hazard ratio; mo., months; NA, not assessable; OS, overall survival.

**Table 6:** Toxicity, according to the CTCAE 4.03 criteria, and dose reductions of ICE + RHT. Dose information was available for 106 patients (53 with locally advanced and 53 with metastatic disease).

	Locally advanced disease (LA-STS, *n* = 54)	Metastatic disease (M-STS, *n* = 56)
Hematological toxicity	No. of patients (%)	No. of patients (%)
Grade III	11 (20.4)	13 (23.2)
Grade IV	25 (46.3)	20 (35.7)
Fever in cytopenia	15 (27.8)	12 (21.4)
Therapy-related deaths	3 (5.6)	0 (0.0)

ICE chemotherapy doses		
Patients never receiving full dose	30 (55.6)	31 (55.4)
Further dose reductions needed (compared to dose of first applied cycle)	23 (42.6)	32 (57.1)
No information on dosing available	1 (1.8)	3 (5.3)

## Data Availability

The data used to support the findings of this study are available from the corresponding author upon request.

## Supplementary Materials

Supplementary Materials Supplementary Figure 1: selected patient populations. Abbreviations: STS, soft-tissue sarcoma. Supplementary Figure 2: relative dose intensity of applied ICE cycles. Full-dosed ICE (as described in the patients and methods paragraph) over four days was considered 100%. Supplementary Table 1: prognostic factors for progression-free survival for LA-STS patients. *p* values were calculated using the log-rank test for univariate analysis. For multivariate analysis using a Cox model, only parameters with a *p* < 0.10 in univariate analyses were considered. Abbreviations: HR, hazard ratio; mo, months; NA, not assessable; OS, overall survival. Supplementary Table 2: prognostic factors for overall survival for M-STS patients. *p* values were calculated using the log-rank test for univariate analysis. For multivariate analysis using a Cox model, only parameters with a *p* < 0.10 in univariate analyses were considered. Abbreviations: HR, hazard ratio; mo, months; NA, not assessable; OS, overall survival.
