# Supplementary material for: Ifosfamide, Carboplatin, and Etoposide (ICE) in Combination with Regional Hyperthermia as Salvage Therapy in Patients with Locally Advanced Nonmetastatic and Metastatic Soft-Tissue Sarcoma
Source: Sarcoma. 2020 Feb 27;2020:6901678. doi: 10.1155/2020/6901678 (PMC7068144; doi:10.1155/2020/6901678)
Supplement: Supplementary Materials — Supplementary Figure 1: selected patient populations. Abbreviations: STS, soft-tissue sarcoma. Supplementary Figure 2: relative dose intensity of applied ICE cycles. Full-dosed ICE (as described in the patients and methods paragraph) over four days was considered 100%. Supplementary Table 1: prognostic factors for progression-free survival for LA-STS patients. p values were calculated using the log-rank test for univariate analysis. For multivariate analysis using a Cox model, only parameters with a p < 0.10 in univariate analyses were considered. Abbreviations: HR, hazard ratio; mo, months; NA, not assessable; OS, overall survival. Supplementary Table 2: prognostic factors for overall survival for M-STS patients. p values were calculated using the log-rank test for univariate analysis. For multivariate analysis using a Cox model, only parameters with a p < 0.10 in univariate analyses were considered. Abbreviations: HR, hazard ratio; mo, months; NA, not assessable; OS, overall survival. [file 6901678.f1.pdf]

Supplementary material

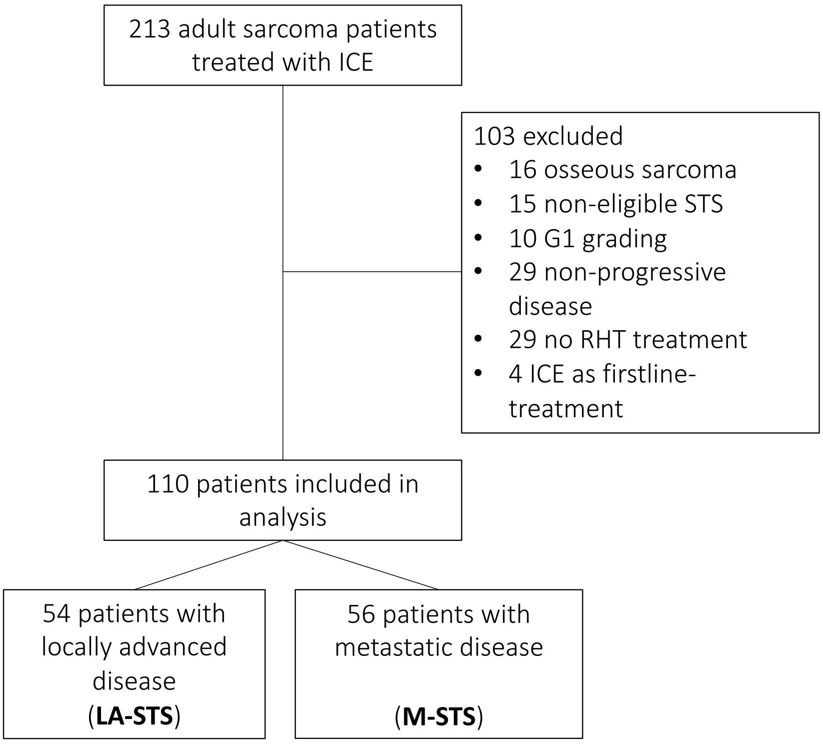

Supplementary Figure 1: Selected patient populations. **Abbreviations:** STS: soft-tissue sarcoma

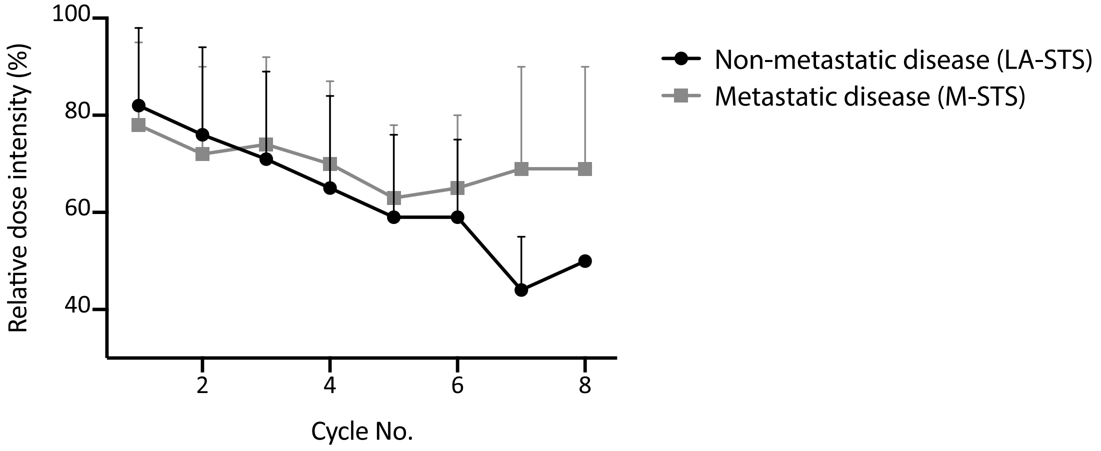

Supplementary Figure 2: Relative dose intensity of applied ICE cycles. Full-dosed ICE (as described in the patients and methods paragraph) over four days was considered 100%.

Supplementary Table 1: Prognostic factors for progression-free survival for LA-STS patients. P values were calculated using the log-rank test for univariate analysis. For multivariate analysis using a Cox model, only parameters with a p <0.10 in univariate analyses were considered. **Abbreviations:** HR: Hazard ratio, mo: months, NA: not assessable, OS: overall survival.

Parameter	N	Univariate analysis		Cox model	
		Median PFS, mo (95% CI)	p	HR (95% CI)	p
**Age**			0.29		
**>50 years**	38	10 (8-15)			
**≤50 years**	16	9 (6-29)			
**Sex**			0.49		
**Male**	31	10 (8-12)			
**Female**	23	9 (6-22)			
**Treatment line**			0.15		
**2nd line**	46	10 (9-15)			
**> 2nd line**	8	9 (6-NA)			
**Grading**			**0.003**	3.10 (1.028-9.34)	**0.045**
**3**	32	9 (5-10)			
**2**	22	20 (11-36)			
**Histologic sub-entity**			**0.043**	0.88 (0.395-1.98)	0.762
**Lipo-/Leiomyosarcoma**	28	11 (6-29)			
**Non-Lipo-/Leiomyosarcoma**	26	9 (7-11)			
**Surgery**			**0.008**	0.45 (0.196-1.02)	0.054
**Yes**	31	11 (9-21)			
**No**	23	8 (5-11)			
**Response/Disease stabilization as best radiographic response with ICE+RHT**			**<0.0001**	0.15 (0.054-0.39)	**<0.001**
**Yes**	26	10 (9-27)			
**No**	9	3 (2-NA)			
**Type of progression**			**0.0002**	0.42 (0.175-0.99)	**0.048**
**Progression after response to prior chemotherapy**	32	11 (9-27)			
**Primary refractory disease**	22	5 (4-10)			

Supplementary Table 2: Prognostic factors for overall survival for M-STS patients. P values were calculated using the log-rank test for univariate analysis. For multivariate analysis using a Cox model, only parameters with a p <0.10 in univariate analyses were considered. **Abbreviations:** HR: Hazard ratio, mo: months, NA: not assessable, OS: overall survival.

**Parameter**	**N**	**Univariate analysis**		**Cox model**	
		**Median OS, mo (95% CI)**	**p**	**HR (95% CI)**	**p**
**Age**			0.24		
**>50 years**	27	15 (11-27)			
**≤50 years**	29	11 (8-19)			
**Sex**			0.56		
**Male**	26	11 (7-19)			
**Female**	30	13 (10-31)			
**Treatment line**			0.12		
**2nd line**	40	11 (8-17)			
**> 2nd line**	16	15 (11-42)			
**Grading**			0.07	1.33 (0.68-2.60)	0.406
**3**	39	10 (7-19)			
**2**	17	15 (13-NA)			
**Histologic sub-entity**			0.19		
**Lipo-/Leiomyosarcoma**	22	11 (7-17)			
**Non-Lipo-/Leiomyosarcoma**	34	17 (11-34)			
**Surgery**			0.52		
**Yes**	15	15 (11-35)			
**No**	41	11 (7-19)			
**Response/Disease stabilization as best radiographic response with ICE+RHT**			**<0.0001**	0.36 (0.19-0.70)	**0.002**
**Yes**	26	18 (15-35)			
**No**	24	7 (5-19)			
**Type of progression**			0.33		
**Progression after response to prior chemotherapy**	19	15 (11-34)			
**Primary refractory disease**	37	10 (7-17)			
